# Use of diagnostic coronary angiography in women and men presenting with acute myocardial infarction: a matched cohort study

**DOI:** 10.1186/s12872-016-0248-9

**Published:** 2016-06-01

**Authors:** Louise Hougesen Bjerking, Kim Wadt Hansen, Mette Madsen, Jan Skov Jensen, Jan Kyst Madsen, Rikke Sørensen, Søren Galatius

**Affiliations:** Department of Cardiology, University Hospital Gentofte, Kildegårdsvej 28, 2900 Hellerup, Denmark; Department of Cardiology, University Hospital Bispebjerg, Copenhagen, Denmark; Department of Public Health, University of Copenhagen, Copenhagen, Denmark; Department of Clinical Medicine, University of Copenhagen, Copenhagen, Denmark; Emergency Department, Holbaek Hospital, University of Copenhagen, Holbaek, Denmark

**Keywords:** Acute myocardial infarction, Coronary angiography, Cardiac catheterization, Gender

## Abstract

**Background:**

Based on evident sex-related differences in the invasive management of patients presenting with acute myocardial infarction (AMI), we sought to identify predictors of diagnostic coronary angiography (DCA) and to investigate reasons for opting out an invasive strategy in women and men.

**Methods:**

The study was designed as a matched cohort study. We randomly selected 250 female cases from a source population of 4000 patients hospitalized with a first AMI in a geographically confined region of Denmark from January 2010 to November 2011. Each case was matched to a male control on age and availability of cardiac invasive facilities at the index hospital. We systematically reviewed medical records for risk factors, comorbid conditions, clinical presentation, and receipt of DCA. Clinical justifications, as stated by the treating physician, were noted for the subset of patients who did not receive a DCA.

**Results:**

Overall, 187 women and 198 men received DCA within 60 days (75 % vs. 79 %, hazard ratio: 0.82 [0.67-1.00], *p* = 0.047).In the subset of patients who did not receive a DCA (*n* = 114), clinical justifications for opting out an invasive strategy was not documented for 21 patients (18.4 %). Type 2 myocardial infarction was noted in 11 patients (women versus men; 14.5 % vs. 3.8 %, *p* = 0.06) and identified as a potential confounder of the sex-DCA relationship.

Receipt of DCA was predicted by traditional risk factors for ischaemic heart disease (family history of cardiovascular disease, hypercholesterolemia, and smoking) and clinical presentation (chest pain, ST-segment elevations). Although prevalent in both women and men, the presence of relative contraindications did not prohibit the use of DCA.

**Conclusion:**

In this matched cohort of patients with a first AMI, women and men had different clinical presentations despite similar age. However, no differences in the distribution of relative contraindications for DCA were found between the sexes. Type 2 MI posed a potentiel confounder for the sex-related differences in the use of DCA. Importantly,clinical justification for opting out an invasive strategy was not documented in almost one fifth of patients not receiving a DCA.

**Electronic supplementary material:**

The online version of this article (doi:10.1186/s12872-016-0248-9) contains supplementary material, which is available to authorized users.

## Background

Ischemic heart disease (IHD) constitutes the leading cause of years of life lost worldwide and is one of the leading causes of death in both women and men [1]. Despite recommendations for similar treatment of women and men presenting with acute myocardial infarction (AMI) by the European Society of Cardiology and the Danish Society of Cardiology [2, 3], differences in invasive management of women and men have been widely reported [4–7]. The majority of studies have compared men and women with AMI at different ages prohibiting an appropriate comparison of baseline characteristics and comorbidities. Moreover, it has not been properly investigated whether clinical justifications for opting out an invasive strategy in patients presenting with AMI differ between women and men. The objective of this study was to characterize an age-matched cohort of women and men hospitalized with AMI, and to investigate reasons for opting out an invasive treatment strategy in a real-world setting.

## Methods

### Design overview

**Fig. 1:**
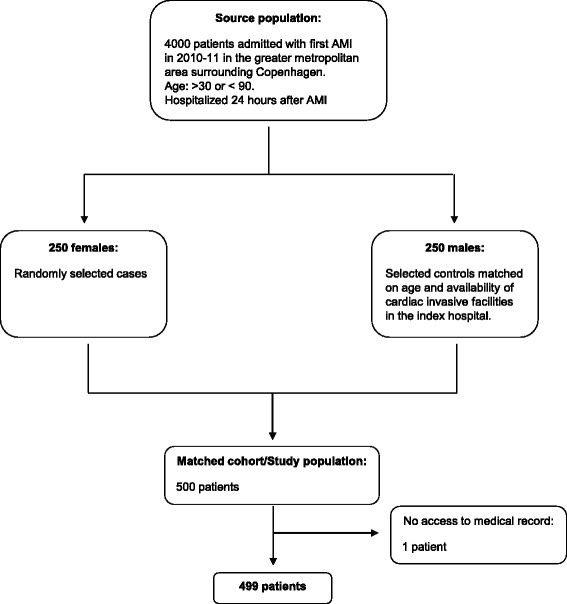
Flowchart. Flow chart showing the selection of the study population from the source population

This study was designed as a matched cohort study. Using all patients hospitalized between 1 January 2010 and 2 November 2011 with a first AMI in the Greater metropolitan area surrounding Copenhagen (*n* = 4000) as our source population, we randomly selected 250 female cases and matched them in a 1:1 ratio with 250 male controls based on age and availability of cardiac invasive facilities in the index hospital. This matched cohort of 500 patients constituted our *study population* (Fig. 1) for which we conducted a systematic, retrospective collection of patient data from medical records. Patients were followed for 60 days. We identified predictors of receiving a cardiac catheterization during follow-up, frequencies of relative contraindications for coronary angiography, and documented reasons for opting out an invasive treatment strategy.

### Settings

The greater metropolitan area surrounding Copenhagen included 10 hospitals with medical admission wards plus two high-volume hospitals with invasive heart centers performing diagnostic coronary angiography (DCA), percutaneous coronary intervention (PCI) and coronary artery bypass graft surgery (CABG). The hospital catchment areas covered the greater northern capital area and a smaller remote island (Bornholm); a total of 1.68 million inhabitants in 2010 [8]. Pre-hospital triage ensured direct transport of patients presenting with ST-segment elevation myocardial infarction (STEMI) to treatment with primary PCI at an invasive heart center. Non-ST-segment elevation myocardial infarction (NSTEMI) patients were in general initially treated at the nearest hospital and subsequently referred to an invasive heart center for further invasive assessment. The guidelines for treatment of AMI issued by the Danish society of Cardiology follow those of European Society of Cardiology [9] and explicitly state that, unless contraindicated, all patients with AMI irrespective of sex should be offered a DCA.

### Patients

We identified a source population of patients hospitalized with a first AMI from 1 January 2010 to 2 November 2011 from the Danish National Patient Register, which contains information on all hospital admissions in Denmark since 1978 [10]. The International Classification of Diseases (ICD) 10^th^ revision codes for AMI (I21-I21.9) in the Danish National Patient Register have previously been validated showing a positive predictive value of 93.5 % [11]. We linked data on dates of index admission, index hospital, and cardiac procedures to information on dates of death from the Danish Register of Causes of Death and demographics from the Danish Civil Registration System using the unique personal civil registration number provided to all Danish citizens at birth or immigration. Further restrictions to the source population were applied; Patients discharged on the day of admission were excluded, as they were unlikely to have experienced an actual myocardial infarction. Similarly, death on the day of admission rendered patients ineligible for invasive treatment and thus were excluded. Finally, we excluded patients younger than 30 years, since AMI in this age group are rarely related to atherosclerosis, and patients older than 90 years, due to frailty in this elderly group [12–14].

From the source population a random draw of 250 female cases matched with 250 male controls of similar age and similar access to invasive cardiac treatment at index hospital constituted the study population. The matching procedure was performed using the *MatchIt* package [15] of statistical software R, version 3.1.0 [16].

### Data collection

Patient-level clinical data was collected from electronic medical records. The collection process was standardized using pretested extraction sheets (Additional file 1: Extraction sheet) in order to ensure consistent and comparable data. Each extraction sheet was divided into seven main topics: 1) index admission, 2) risk factors, 3) comorbidities, 4) electrocardiographic (ECG) findings, 5) clinical presentation, 6) in-hospital medications, and 7) blood test results. Only information available to the treating physicians prior to any cardiac catheterization was collected; in patients who did not receive a DCA all information from the hospital stay was collected. Data was entered in an electronic database and compiled with register-based data using the personal civil registration number as described above. Data collection, analysis and interpretation were performed by one specially trained individual (LHB) to ensure consistency and reproducibility. Upon completing the initial data collection process, the process was repeated for the initial 80 medical records and compared the obtained data in order to address potential intra-observer variability.

### Contextual variables

For each patient not receiving a coronary angiography we noted the clinical justification, as stated in the medical record by the treating physician, word-for-word and categorized them into 11 arbitrarily defined groups: DCA already performed, death before DCA, DCA declined by patient, DCA not possible to perform or not indicated, comorbidities/bad habitual conditions, lack of symptoms, type 2 MI, high age, DCA not mentioned in the medical record, no AMI, and other. The diagnosis of type 2 MI was assigned when one of two conditions was met: (1) the treating physician documented the qualifying AMI event as a type 2 myocardial infarction directly in the medical records, or (2) the reasons stated by the treating physician for opting out a DCA were consistent with criteria listed in the international definition of type 2 MI [17]. The definitions of relative contraindications for cardiac catheterization were based on guidelines developed by the Danish Society of Cardiology [18]. Uncontrolled hypertension, fever or active infection, malignant or terminal disease, risk of bleeding, ongoing bleeding, moderate to severe heart failure, previous allergy to contrast, digoxin intoxication, and electrolyte disturbances were all considered as individual relative contraindications for DCA. We used data collected from electronic medical records for quantifying the distribution of these relative contraindications in the study population. We defined *uncontrolled hypertension* as an elevated systolic blood pressure (SBP) > [180 mmHg] or diastolic blood pressure (DBP) > [110 mmHg]). *Fever or active infection* was defined as a white blood cell count (WBC) above 8.8 x 10^9^/liter or temperature more than 38 degrees Celsius. *Malignant or terminal disease* was defined as severe anemia with hemoglobin below 6 mM or renal failure with serum-creatinine above 250 mM. *Risk of bleeding* was defined as a platelet count below 145 μM or an International Normalized Ratio (INR) above 1.2, and *moderate to severe heart failure* as a history of heart failure, or clinical findings of neck vein distension, dependent edema, or pulmonary edema. *Electrolyte disturbances* were defined as potassium levels above 4.6 or below 3.5 mM.

All collected ECG findings and blood test results were those available to the treating physician as upon hospitalization; but always prior to the time of cardiac catheterization in patients receiving a DCA. The only exception was the second measurement of troponins (“*troponin II”*) and the highest troponin value measured during hospitalization (“*peak troponin”*) which was sometimes only available subsequent to a coronary angiography. As different troponin assays were used across hospitals we standardized all troponin-levels against the upper reference limit to enable comparisons.

### Statistical methods

We present discrete data as counts and percentages, and continuous data as median and interquartile range (IQR). Categorical data were compared using a Chi-squared test or, if the expected number of observations in a group were less than five, using Fisher’s exact test. Continuous data were analyzed using the non-parametric Mann-Whitney *U*-test. In order to identify predictors of DCA we constructed uni- and multivariable logistic regression models with receipt of coronary angiography within 60 days as the dependent variable and patient characteristics as independent variables. The multivariable logistic regression model was build using a backwards stepwise procedure using a *p*-value of 0.10 as cutoff for inclusion. The final model was tested for collinearity and interactions. Time-to-event analyses of all-cause death and receipt of DCA were conducted using proportional hazards Cox regressions. The assumption of proportional hazards was assessed with log-log curves and by testing the Schoenfeld residuals for time-dependency. Assumptions were found valid. All statistical tests had a two-sided significance level of 0.05. The analyses were conducted using Stata Statistics/Data analysis, MP 14.0 StataCorp, Texas, USA.

## Results

The matching procedure successfully balanced the 250 women and 250 men on age and type of index hospital (Additional file 2: Table S1). The study population contained more elderly patients than the source population as expected from the use of female cases. Complete medical records were available for 499 patients (Fig. 1). Table 1 shows baseline characteristics of the study population. A higher proportion of women had heart failure and a family history of cardiovascular diseases (CVD) compared to men. In contrast, more men than women had known ischemic heart disease (IHD), prior PCI, and prior CABG. Numerically, men were more likely to have chest pain than women, whereas more women presented with atypical symptoms such as nausea and vomiting. The only significant difference in ECG patterns was a higher proportion of ST-depressions among men compared to women, although a tendency toward a higher rate of left bundle branch block (LBBB) among women was apparent. Women presented with higher systolic blood pressure, and heart rate but lower serum-creatinine levels than men. Coronary angiography was performed in 385 patients (77.2 %) within 60 days of index hospitalization; 198 men and 187 women. Thus, the cumulative incidence of DCA at 60 days was higher for men than women (79.2 % vs. 75.1 %, HR 0.82 [0.67-1.00], *p* = 0.047). There was no significant difference in all-cause mortality at 60 days between women and men (9.6 % vs. 10.4 %, HR 0.91 [0.52-1.59], *p* = 0.74), even when separated into age-quartiles (Additional file 3: Table S2).

**Table 1:** Baseline characteristics and clinical presentations

	Study Cohort	Women	Men	*p*-value
Number	*n* = 499	*n* = 249	*n* = 250	*n* = 499
Age *median(IQR)*		74 (62-81)	74 (62-81)	0.96
Admission to invasive heart center		89 (35.6)	92 (36.8)	0.78
Risk factors				
Family history of CVD		80 (32.1)	61 (24.4)	0.06
Arterial hypertension		122 (49.0)	119 (47.6)	0.76
Diabetes mellitus		36 (14.5)	39 (15.6)	0.72
Hypercholesteroleamia		80 (32.1)	85 (34.0)	0.7
Smoking		69 (27.7)	82 (32.8)	0.08
Prior PCI		6 (2.4)	16 (6.4)	0.047
Prior CABG		6 (2.4)	21 (8.4)	0.005
Previous MI		9 (3.6)	9 (3.6)	0.99
Co-morbidities				
Heart-related				
Known IHD		20 (8.0)	46 (18.4)	0.001
Heart failure		35 (14.1)	19 (7.6)	0.020
Valvular heart disease		26 (10.4)	19 (7.6)	0.27
Atrial fibrillation		39 (15.7)	40 (16.0)	0.92
Other				
COPD		23 (9.2)	27 (10.8)	0.56
PAOD		14 (5.6)	20 (8.0)	0.29
Renal failure		11 (4.4)	9 (3.6)	0.66
Neoplasia		4 (1.6)	12 (4.8)	0.07
Liver failure		0 (0.0)	1 (0.4)	1.00
Stroke		25 (10.0)	31 (12.4)	0.40
Symptoms				
Chest pain		189 (75.9)	203 (81.2)	0.15
Dyspnea		96 (38.6)	81 (32.4)	0.15
Neck pain		25 (10.0)	21 (8.4)	0.53
Diaphoresis		30 (12.1)	36 (14.4)	0.44
Nausea/vomiting		47 (18.9)	31 (12.4)	0.046
Fatigue		7 (2.8)	8 (3.2)	0.8
Abdominal pain		14 (5.6)	8 (3.2)	0.19
Back pain		27 (10.8)	21 (8.4)	0.36
Cardiac arrest		15 (6.0)	14 (5.6)	0.84
Other competing acute conditions at admission?^a^		42 (16.9)	34 (13.6)	0.31
ECG				
ST-elevations		104 (41.8)	107 (42.8)	0.82
ST-depressions		59 (23.7)	86 (34.4)	0.008
LBBB		24 (9.6)	16 (6.4)	0.18
Q-wave		37 (14.9)	38 (15.2)	0.92
Systolic blood pressure^e^		140 (126-160)	137 (119.5-155)	0.013
Heart rate^e^		88 (70-105)	81 (66-97)	0.008
Troponin level I^b,c,e^		3.2 (1.4-9.2)	2.5 (1.0-8.4)	0.25
Troponin level II^b,d,e^		4.5 (2.1-12.5)	6.1 (2.0-35)	0.14
Peak troponin level^b,e^		12.4 (4.7-44.3)	20.3 (4.1-73.6)	0.23
Creatinine level^e^		70 (59-86.5)	87 (74-100)	<0.001

Numbers are counts (%) unless otherwise stated. Numbers of missing values varied from 45 (Systolic blood pressure) to 269 (troponine concentration II). *IQR* interquartile range, *CVD* cardiovascular disease, *PCI* percutaneous coronary intervention, *CABG* coronary artery bypass graft surgery, *AMI* acute myocardial infarction, *IHD* Ischemic heart disease, *COPD* chronic obstructive pulmonary disease, *PAOD* peripheral arterial occlusive Disease, *LBBB* left bundle branch block, *INR* International normalized ratio. ^a^Competing acute conditions include infections, dementia, ileus etc. ^b^Standardized against upper limit. ^c^The first troponin value measured before CAG, ^d^the second troponin value measured before CAG. ^e^median (IQR)

In terms of relative contraindications for DCA women were more likely to have electrolyte-disturbances than men (Table 2). Compared to men, there was a trend towards more cases of uncontrolled hypertension among women, as well as a higher proportion of women with at least one relative contraindication. Among the two most common relative contraindications, uncontrolled hypertension, and risk of bleeding, no sex-related differences were found. The presence of relative contraindications did not preclude the use of DCA.

**Table 2:** Relative contraindications as defined by national guidelines

	Female	Male	*p*-value
Uncontrolled hypertension	29 (13.1)	18 (8.0)	0.08
Fever or active infection	0 (0.0)	1 (1.47)	0.5
Malignant or terminal disease	11 (5.5)	4 (2.2)	0.12
Risk of bleeding	23 (14.7)	26 (17.3)	0.54
Ongoing bleeding	5 (2.0)	9 (3.6)	0.42
Moderate/severe heart failure	8 (3.2)	5 (2.0)	0.6
Previous allergy to contrast	0(0.0)	0(0.0)	NA
Digoxin intoxication	0 (0.0)	0 (0.0)	NA
Electrolyte-disturbances (4.6 mM < potassium level < 3.5 mM)	49 (24.6)	29 (15.8)	0.031
At least one of the above mentioned relative contraindication (excluding heart failure)	130 (52.0)	120 (48.0)	0.060

Numbers are counts (%) unless otherwise stated

We used clinical data to quantify the distribution of relative contraindications for men and women. The contraindications were not necessarily listed directly by the physicians in the medical records

**Fig. 2:**
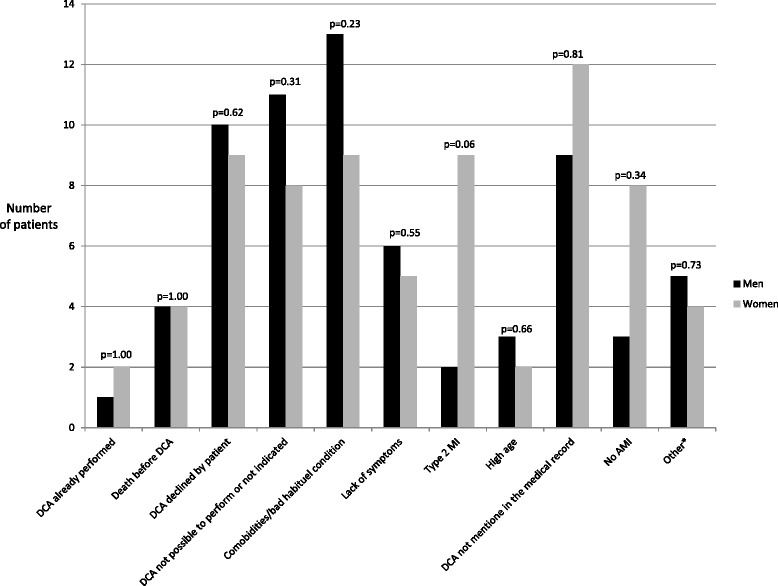
Reasons for omitting DCA stated in the medical records by the treating physicians. The y-axis indicates number of patients. It was possible for the patients to be categorized in more than one of the groups. 23 patients were categorized in two groups, 1 patient was categorized in three groups. *This category includes risk of bleeding due to vitamin K antagonist treatment, pace maker implantation of higher priority than DCA, etc

Table 3 displays univariable predictors of DCA at 60 days. Admission to a hospital with invasive cardiac facilities, known family history of CVD, hypertension, hypercholesterolemia, smoking, and chest pain were all associated with a higher use of DCA. On the other hand, previous CABG, valvular heart disease, atrial fibrillation, COPD, renal failure, stroke, dyspnea, and abdominal pain were associated with less use of DCA. After multivariable analysis arterial hypertension, hypercholesterolemia, smoking, known IHD, chest pain, and ST-elevation persisted as significant positive predictors of DCA. Age, prior CABG, COPD, renal failure, stroke, and Q-wave were negative predictors of DCA (Table 4). In total 114 patients (22.9 %) did not receive a DCA; 52 men and 62 women. Clinical justifications for opting out an invasive treatment strategy, as stated by the treating physician, are summarized in Fig. 2. Most frequent reasons were multiple comorbidities or poor habitual condition (19.3 %), patients who declined invasive examination (16.7 %), or DCA not deemed feasible or indicated (16.7 %). Notably, in 21 (18.4 %) of the cases no reason at all for opting out a coronary angiography was documented in the medical record by the treating physician. There were no significant sex-related differences in any of the 11 groups, but a trend towards more cases of type 2 MI in women compared to men (14.5 % vs. 3.8 %, *p* = 0.06).

**Table 3:** Univariable predictors of receipt of DCA at 60 days

	OR	95 % CI	*p*-value
Female	0.79	0.52-1.20	0.28
Age			
< 60 years	Reference		
60-69 years	1.26	0.33-4.84	0.73
70-79 years	0.26	0.1-0.72	<0.001
≥ 80 years	0.04	0.02-0.10	<0.001
Admission to a hospital with invasive cardiac facilities	4.23	2.43-7.36	<0.001
Risk factors			
Family history of CVD	6.99	3.31-14.79	<0.001
Arterial hypertension	1.67	1.09-2.55	0.019
Diabetes mellitus	0.72	0.42-1.26	0.25
Hypercholesteroleamia	4.21	2.36-7.52	<0.001
Smoking	1.91	1.45-2.52	<0.001
Prior PCI	3.07	0.71-13.33	0.14
Prior CABG	0.41	0.18-0.90	0.027
Previous AMI	1.5	0.43-5.28	0.53
Co-morbidities			
Heart-related			
Known IHD	1.12	0.59-2.10	0.73
Heart failure	0.67	0.36-1.25	0.21
Valvular heart disease	0.40	0.21-0.76	0.005
Atrial Fibrillation	0.36	0.21-0.60	<0.001
Other			
COPD	0.33	0.18-0.60	<0.001
PAOD	0.81	0.37-1.79	0.60
Renal failure	0.11	0.04-0.30	<0.001
Neoplasia	0.88	0.28-2.80	0.84
Liver failure	-	-	-
Stroke	0.19	0.10-0.33	<0.001
Symptoms			
Chest pain	5.64	3.53-9.00	<0.001
Dyspnea	0.47	0.31-0.72	0.001
Neck pain	0.94	0.46-1.91	0.86
Diaphoresis	1.01	0.54-1.87	0.98
Nausea/vomiting	0.71	0.41-1.23	0.22
Fatigue	0.81	0.25-2.59	0.72
Abdominal pain	0.19	0.08-0.45	<0.001
Back pain	1.54	0.70-3.38	0.29
Cardiac arrest	0.76	0.33-1.78	0.53
ECG			
ST-elevations	5.36	3.12-9.21	<0.001
ST-depressions	0.69	0.44-1.08	0.108
LBBB	0.52	0.26-1.03	0.06
Q-wave	0.62	0.36-1.06	0.08
Other			
Systolic BP	1.01	1.00-1.02	0.024
Diastolic BP	1.02	1.01-1.04	0.002
HR	0.99	0.98-1.00	0.010
Troponine 1	0.99	0.99-1.00	0.028
Troponine 2	1.00	1.00-1.00	0.34
Peak troponine	1.00	1.00-1.00	0.59
Creatinine	0.98	0.97-0.99	<0.001

*CVD* cardiovascular disease, *PCI* percutaneous coronary intervention, *CABG* coronary artery bypass graft surgery, *AMI* acute myocardial infarction, *IHD* Ischemic heart disease, *COPD* Chronic obstructive pulmonary disease, *PAOD* Peripheral Arterial Occlusive Disease, *LBBB* left bundle branch block, *ECG* Electrocardiogram

**Table 4:** Multivariable predictors of receipt of DCA at 60 days

	OR	95 % CI	*p*-value
Age	0.90	0.87-0.94	<0.001
Admission to center	2.8	1.27-6.21	0.011
Family history of CVD	2.35	0.92-5.97	0.072
Arteriel hypertension	2.38	1.23-4.62	0.010
Hypercholesteroleamia	3.00	1.38-6.48	0.005
Smoking	2.03	1.39-2.97	<0.001
Prior CABG	0.25	0.06-0.98	0.047
Co-morbidities			
Heart-related			
Known IHD	3.85	1.27-11.63	0.017
Other			
COPD	0.37	0.15-0.87	0.023
Renal failure	0.20	0.05-0.83	0.027
Stroke	0.31	0.13-0.70	0.005
Symptoms			
Chest pain	2.99	1.58-5.67	0.001
ECG			
ST-elevations	4.44	2.05-9.60	0.000
Q-wave	0.35	0.15-0.78	0.011

*CABG* coronary artery bypass graft surgery, *COPD* Chronic obstructive pulmonary disease, *CVD* cardiovascular disease, *ECG* Electrocardiogram, *IHD* Ischemic heart disease

## Discussion

### Key findings

This study used detailed clinical information from 500 medical records of patients hospitalized with a first AMI to investigate the clinical basis for referring men and women to DCA. Classical risk factors, symptoms and clinical findings predicted the receipt of DCA in this matched cohort. Women had an 18 % lower risk for receipt of DCA at 60 days than men, but a similar risk for all-cause mortality despite accounting for differences in age and type of index hospital. Surprisingly, no clinical justification for refraining from an invasive treatment strategy was documented in almost one fifth of the records of patients who did not receive a DCA.

### Interpretations

The matched design of our study was intended to address two issues. First, it has been suggested that differences in patient characteristics, treatments and outcomes can largely be attributed to the differences in age between women and men presenting with ischemic heart disease [6]. Second, the use of cardiac catheterization is strongly associated with hospital-availability of this procedure [19]. By matching on age and hospital, these confounding effects were managed prior to our analyses. Despite the somewhat limited power of our analyses, we identified significant differences in patient characteristics between women and men of similar age; i.e. heart failure was more prevalent in women while more men presented with known IHD and prior revascularizations. Based on these findings a sex-related difference in the etiology of AMI seems more plausible than age in explaining previously observed differences in characteristics between women and men [20].

Our logistic and Cox proportional hazards regression analyses yielded differing results in terms of the association between sex and receipt of DCA. The reason for this lies in the poorer power of the logistic regression which only incorporates counts, as compared to the Cox regression modeling time-to-event data. We relied on the results of the latter, as the logistic regression attributes equal weights to early and late procedures and thus does not address the timing of DCAs, which we deemed of clinical relevance. Thus, despite women having a lower risk for DCA at 60 days than men, we were unable to demonstrate any significant differences in the most common relative contraindications. Interestingly, non of the defined relative contraindications were listed by the treating physician as reasons for not performing DCA in any patients. Of note, no patients with contrast allergy or pregnancy were found in our cohort; as these contraindications may be considered more severe. Classical risk factors such as family history of CVD, hypercholesterolemia, and smoking; symptoms of chest pain, and clinical findings of ST-segment elevations were significant predictors of an invasive strategy in our cohort. However, women were more likely than men to present with atypical symptoms of nausea and vomiting. Similar findings have been made in other studies [21, 22] and suggest that increased vigilance is required when examining women in the emergency setting. Use of computed coronary tomography might be an option, as some trials suggest this method is effective for identification of patients in need of an invasive strategy [23].

By reviewing medical records containing the treating physicians’ reflections and rationale for opting out an invasive treatment strategy in some patients, we got a unique insight into the actual treating process of patients with AMI. Noticeably, we found a trend towards more cases of type 2 MI in women and a potential confounding effect on the sex-DCA relationship. This is in accordance with the findings of Saaby et al. who showed a higher prevalence of type 2 MI in women compared to men, and less cardiac catheterizations in type 2 MI [24]. It is possible that type 2 MI plays a larger role in the sex-related differences in treatment of AMI, than previously known.

Prior studies have proposed several hypotheses as to why sex-related differences in the management of AMI exist. It has been discussed if women were more likely to refuse DCA than men. Golden et al. showed that fewer women preferred DCA in the emergency room and in-hospital [25]. Heidenreich et al. found that elderly women were more likely to refuse DCA than men, but the rate of refusals was low (5.1 %) [26]. In another study, Mumma et al. found that female patients were less likely to receive a cardiac catheterization recommended by the physician, yet this could not explain the gender gap [27]. In our study 17 % of those who were not invasively investigated had refused DCA, without any sex-related differences. Physicians’ reasons for not adopting an invasive strategy in women compared to men has previously been investigated [28], but no study examining this issue based on medical records in a real life setting is known by us. Although evidence and guidelines supports that all patients with AMI should undergo DCA, perhaps with the exception of low risk biomarker positive women [29], cases where lack of evidence drives to omitting DCA in patients with AMI exist. According to Poon et al. significantly more women than men were not referred for DCA because the physician found that it was not supported by evidence [28]. In our study the decision not to refer to a coronary angiography was justified in more than 80 % of the cases, but in the remainder of patients an assessment of indications for cardiac catheterization was not provided. Interestingly, women were more prevalent in this subset of patients. This finding emphasizes the importance of considering and documenting clinical decisions; especially when deviating from guideline-recommended treatments.

### Strengths and limitations

Our study included detailed data from medical records representing the actual information available to the treating physicians. This provided unique insights to the clinical decision underlying referral to cardiac catheterization in a real world-setting.

Our study has some important limitations. First, this was an observational study prohibiting any conclusion regarding causality. Second, given the retrospective data collection process some degree of misclassification cannot be ruled out. Hence, contradictory or inconsistent descriptions in the medical records may have resulted in misinterpretation or missing. We addressed this issue by checking reproducibility through standardized extraction sheets, a specially trained data collector and extensive rereading of the first 80 patients medical records. Third, we did not have information on the level of training or specialization of the treating physicians; particularly the physician who decided whether or not the patient should receive an invasive treatment strategy. Finally, the sample size was small and the study thus underpowered to detect significant differences in the subset analyses of clinical justifications. However, logistic and practical constraints made it impossible to include more than 500 patients.

## Conclusion

In this contemporary matched cohort of patients hospitalized with a first AMI we found that patient characteristics differed between women and men despite similar age. Although women had a lower risk for DCA at 60 days than men, we were unable to detect any differences in the distribution of relative contraindications for coronary angiography between the sexes. In patients not referred for DCA, physicians did not document any reasons for opting out this procedure in one fifth of patients. Thus, physicians should focus on managing both women and men in accordance with current guidelines and only refrain from using DCA when evidence-driven. Finally, type 2 MI poses a potential confounder for the sex-DCA relationship and merits further investigations.

### Ethics approval and consent to participate

This project was carried out in accordance with current rules of ethics and legislature. It was approved by The Danish Data Protection Agency [record number 2007-58-0015] and the Danish Health and Medicines Authority [record number 3-3013-376/1/]. The approval from the Danish Health and Medicines Authority provided statutory authority for collecting patient information from all 500 medical records without obtaining written informed consent. All personal information was anonymized upon database closure using a positive integer ranging from 1 to 500 as unique patient identifiers and stored on a secure encrypted hard drive. The conversion key was kept on a separate encrypted hard drive. Register-based studies do not require approval from an Ethics Committee in Denmark.

## Additional files

Additional file 1: Extraction sheet. (PDF 86 kb) Additional file 2: Table S1. (PDF 96 kb) Additional file 3: Table S2. (PDF 330 kb)

## Abbreviations

AMI: acute myocardial infarction
CABG: coronary artery bypass graft
CVD: cardiovascular disease
DBP: diastolic blood pressure
DCA: diagnostic coronary angiography
ECG: electrocardiogram
HR: hazard ratio
ICD: the international classification of diseases
IHD: ischemic heart disease
INR: international ratio
IQR: interquartile range
LBBB: left bundle branch block
NSTEMI: non ST-elevation myocardial infarction
PCI: percutaneous intervention
SBP: systolic blood pressure
STEMI: ST-elevation myocardial infarction
WBC: white blood cell count
