# Supplementary material for: Use of diagnostic coronary angiography in women and men presenting with acute myocardial infarction: a matched cohort study
Source: BMC Cardiovasc Disord. 2016 Jun 1;16:120. doi: 10.1186/s12872-016-0248-9 (PMC4888313; doi:10.1186/s12872-016-0248-9)
Supplement: Additional file 1: — Extraction sheet. (PDF 86 kb) [file 12872_2016_248_MOESM1_ESM.pdf]

Data	Patient 1	Patient 2	Patient 3	Patient 4	Patient 5
1) Index admission					
Personal civil registration number					
Date of index admission					
Sex					
Age					
Index hospital					
Way of admission					
If transferred: Date of first admission					
Diagnostic coronoary angiography (DCA) performed?					
Date for DCA					
Indication for DCA					
Admission diagnosis					
Working diagnosis of acute coronary syndrome?					
If no, then what diagnosis?					
Time from presentation to admission					
Procedure before index admission?					
DCA?					
Date for DCA					
PCI?					
Date for PCI					
CABG?					
Date for CAGB					
DCA performed at this admission?					
Date for DCA					
DCA declination by patient?					
DCA deemed contraindicated by physician?					
Reason/why?					
Final primary diagnosis					
Ongoing pregnancy					
2) Risk factors					
Family history of cardiovascular disease					
Hypertension					
Diabetes mellitus					
Hypercholesterolaemia					
Smoking					
Prior percutaneous intervention (PCI)					
Prior Coronary artery bypass					

graft (CABG)					
Prior myocardial infarction (MI)					
3) Comorbidities					
Known ischeamic heart disease					
Heart failure					
Valvular disease					
Atrial Fibrillation					
Peripheral arterial occlusive Disease (COPD)					
Peripheral arterial occlusive Disease (PAOD)					
Renal failure					
Neoplasia					
Liver failure					
Stroke					
Known contrast-allergy					
NYHA class					
Terminal comorbid condition					
4) Electronic electrocardiogram (ECG) findings					
ST-elevations					
ST-depressions					
Left bundle branch block (LBBB)					
Q-wave					
5) Clinical presentation					
Chest pain					
Dyspnea					
Neck pain					
Diaphoresis					
Nausea					
Fatigue					
Abdominal pain					
Back pain					
Cardiac arrest					
Other competing acute conditions at admission					
If yes, which?					
Ongoing bleeding					
Where?					
Systolic blood-pressure					
Diastolic blood-pressure					
Heart rate					
Repiratory rate					
Temperature					
Blood saturation					

Body mass index					
Dependent oedema					
Pulmonary oedema					
Neck vein distension					
6) In-hospital medication					
Digoxin treatment					
Metformin treatment					
7) Blood test results					
Hemoglobin					
White blood-cell count (WBC)					
Upper ref. WBC					
Thrombocytes					
Lower ref. thrombocytes					
Upper ref. Thrombocytes					
Creatinine					
Upper ref. Creatinine					
Potassium level					
Upper ref. Potassium					
Lower ref. Potassium					
Troponine concentration I					
Troponine concentration II					
Troponine concentration III					
Upper ref. Troponine					
Troponine I					
CKMB concentration I					
CKMB concentration II					
CKMB concentration III					
Upper ref. CKMB					
International normalised ratio (INR)					
