# Supplementary material for: Use of diagnostic coronary angiography in women and men presenting with acute myocardial infarction: a matched cohort study
Source: BMC Cardiovasc Disord. 2016 Jun 1;16:120. doi: 10.1186/s12872-016-0248-9 (PMC4888313; doi:10.1186/s12872-016-0248-9)
Supplement: Additional file 2: Table S1. — (PDF 96 kb) [file 12872_2016_248_MOESM2_ESM.pdf]

Table S1

Gender difference in the invasive investigation of AMI patients							
	Denmark	Capital area/region*	Men	Women	Study Population	Men	Women
No. of patients	11 418	4000	2641	1359	500	250	250
Age, median (IQR)	*68 (58-78)*	*66 (57-77)*	*64 (55-73)*	*73 (62-82)*	*74 (62-81)*	*74 (62-81)*	*74 (62-81)*
Women, %	35.6	34.0	-	-	50.0	-	-
AMI diagnosis							
STEMI	41.8	36.0	39.6	29.2	30.2	31.2	29.2
NSTEMI	31.2	37.0	35.9	39.1	39.6	39.2	40.0
AMI unspecified	27.0	27.0	24.5	31.7	30.2	29.6	30.8
Admission to center, %	*44.6*	*49.9*	*53.4*	*43.1*	*44.8*	*46.0*	*43.6*
Duration of admission (days)	4 (3-6)	4 (3-6)	4 (3-6)	5 (3-6)	5 (3-6)	5 (3-6)	5 (3-7)
KAG within 60 days, %	78.9	81.8	85.7	74.0	73.8	76.4	71.2
Death within 60 days, %	8.5	7.5	6.0	10.4	10.2	10.4	10.0

Italic lines are propensity score matched variables
**The greater metropolitan area surrounding Copenhagen*
