# Supplementary material for: Use of diagnostic coronary angiography in women and men presenting with acute myocardial infarction: a matched cohort study
Source: BMC Cardiovasc Disord. 2016 Jun 1;16:120. doi: 10.1186/s12872-016-0248-9 (PMC4888313; doi:10.1186/s12872-016-0248-9)
Supplement: Additional file 3: Table S2. — (PDF 330 kb) [file 12872_2016_248_MOESM3_ESM.pdf]

Table S2

n(%)	All	Women	Men	*p*-value
DCA				
1. quartile (age 33-62 years)	120 (95.2)	57 (90.5)	63 (100)	0.012
2. quartile (age 63-75 years)	120 (90.9)	60 (90.9)	60 (90.9)	1.00
3. quartile (age 76-82 years)	99 (77.3)	49 (76.6)	50 (78.1)	0.83
4. quartile (age 83-90 years)	46 (40.7)	25 (43.9)	21 (37.5)	0.49
Death within 60 days				
1. quartile (age 33-62 years)	2 (1.6)	1 (1.6)	1 (1.6)	1.00
2. quartile (age 63-75 years)	8 (6.1)	4 (6.1)	4 (6.1)	1.00
3. quartile (age 76-82 years)	13 (10.2)	7 (10.9)	6 (9.4)	0.77
4. quartile (age 83-90 years)	27 (23.7)	14 (24.6)	13 (22.8)	0.83

All numbers are counts (%)
